# Assessing the Modulatory Effects of tDCS and Acupuncture on Cerebral Blood Flow in Chronic Low Back Pain Using Arterial Spin Labeling Perfusion Imaging

**DOI:** 10.3390/brainsci15030261

**Published:** 2025-02-28

**Authors:** Valeria Sacca, Nasim Maleki, Sveta Reddy, Sierra Hodges, Jian Kong

**Affiliations:** Department of Psychiatry, Massachusetts General Hospital and Harvard Medical School, Charlestown, MA 02129, USA; vsacca@mgh.harvard.edu (V.S.); nmaleki@mgh.harvard.edu (N.M.); sreddy21@mgh.harvard.edu (S.R.); sahodges@mgb.org (S.H.)

**Keywords:** cerebral blood flow (CBF), chronic low back pain (cLBP), motor cortex (M1), pulsed continuous arterial spin labeling (pCASL), transcranial direct current stimulation (tDCS), acupuncture

## Abstract

Background: Both transcranial direct current stimulation (tDCS) and acupuncture are promising methods for managing chronic low back pain (cLBP), however, their underlying mechanisms remain unclear. Methods: To explore the neural mechanisms of tDCS and acupuncture on cLBP, we examined how real and sham tDCS applied to the bilateral motor cortex (M1), combined with real or sham acupuncture, influenced cerebral blood flow (CBF) using pulsed continuous arterial spin labeling (pCASL) imaging. tDCS was administered over six sessions, combined with real or sham acupuncture, over one month. Results: Following real tDCS, we observed increased CBF in the bilateral occipital cortex, precuneus, left hippocampus, and parahippocampal gyrus/posterior cingulate cortex. After sham tDCS, CBF decreased in regions including the bilateral superior parietal lobule, precuneus, bilateral precentral and postcentral gyri, and left angular gyrus. Real acupuncture led to reduced CBF in the bilateral occipital cortex and hippocampus, and left posterior cingulate gyrus, and increased CBF in the right postcentral gyrus, superior parietal lobule, and frontal areas. Sham acupuncture was associated with decreased CBF in the bilateral hippocampus and anterior cingulate gyrus. Conclusions: These results suggest both shared and distinct patterns of CBF changes between real and sham tDCS, as well as between real and sham acupuncture, reflecting mode-dependent effects on brain networks involved in pain processing and modulation. Our findings highlight the different neural circuits implicated in the therapeutic mechanisms of tDCS and acupuncture in the management of cLBP.

## 1. Introduction

Transcranial direct current stimulation (tDCS) is a non-invasive neuromodulation technique commonly used to explore the interaction between brain networks and their potential correlation with clinical outcomes [1,2,3,4,5]. tDCS has also shown promise as a therapeutic tool for managing various neurological conditions [6,7,8,9], with one of its most common applications being in the treatment of chronic pain [10,11,12,13,14,15,16,17].

However, despite its encouraging results in pain relief, the underlying neural mechanisms through which tDCS modulates pain are not yet fully understood. As a result, integrating brain imaging data into tDCS research is crucial to better understand its neural mechanisms and advance its use in treating or managing neurological disorders [18]. Gaining a clearer understanding of how tDCS interacts with specific brain regions and circuits involved in pain perception could help refine treatment protocols, leading to more targeted and effective interventions for individuals suffering from chronic pain.

In recent years, various brain imaging techniques, particularly structural and functional magnetic resonance imaging (MRI), have been extensively used to investigate the potential role of tDCS in treating chronic pain conditions [10,19,20,21,22,23,24]. These studies have provided valuable insights into the brain’s structural and functional changes in response to tDCS, revealing how the stimulation modulates neural activity and connectivity in regions involved in pain processing [21,22,23,24]. Despite this growing interest in tDCS, only a few studies have investigated how tDCS influences cerebral blood flow (CBF), which serves as a close surrogate for neural activity within the brain [2,3,25], and none of these studies have focused on chronic pain populations.

CBF can be measured using arterial spin labeling (ASL), a non-invasive MRI technique that intrinsically tags the incoming arterial blood using magnetic pulses to assess tissue perfusion. This method labels the water in arterial blood before it flows and reaches brain tissue, allowing for the assessment of blood flow dynamics in specific regions [26]. ASL produces two types of images: a “tag” image that reflects the magnetization of the inflowing blood, and a “control” image, which captures static tissue signals but differs in the inflow magnetization. Analyzing the differences between the tag and control images provides detailed information about tissue perfusion. Unlike blood oxygenation level-dependent (BOLD) imaging, which uses changes in blood oxygenation as an indirect measure of neural activity, ASL directly measures blood flow, providing a more accurate representation of brain perfusion and serving as a better proxy for neural activity. Given this advantage, studying how tDCS impacts CBF is particularly important in pain research, as it provides valuable insights into how brain stimulation influences both neural and vascular functions [2,3,25].

tDCS has the potential to alter brain activity and connectivity, and as such it could have significant implications for both understanding the mechanisms underlying various neurological and psychiatric disorders, as well as developing novel therapeutic interventions. Since pain is a complex, multidimensional experience involving extensive and intricate neural circuits and brain regions, understanding how tDCS affects CBF in individuals with chronic pain could offer valuable insights into how this non-invasive stimulation technique modulates neural activity in areas involved in pain processing. In addition, research into how tDCS modulates CBF is particularly important because changes in CBF are closely linked to neuronal activity [27,28]. Understanding these changes could reveal important information into how tDCS could be utilized to target specific underlying neural mechanisms contributing to chronic pain states [29], allowing for personalized treatment protocols and more effective targeting of chronic pain. Measuring CBF changes through ASL also has the advantage of providing clues about individual differences in response to tDCS. For example, tDCS has been shown to alter cortical excitability and connectivity in brain regions that play key roles in pain perception and modulation, such as the prefrontal cortex, anterior cingulate cortex, motor cortex, insula, and basal ganglia [10,19,20,30,31]. By examining how tDCS affects CBF in these and other critical pain-related areas, researchers can better assess the long-term effects of stimulation on pain processing pathways and evaluate its potential as a therapeutic tool for chronic pain management and rehabilitation of the dysfunctional circuits in chronic pain.

Few studies in the literature have characterized the changes in CBF induced by tDCS [2,3,25]. Zheng et al. [2] found that different modes of tDCS applied to the right motor cortex were associated with varying CBF changes in the brain in a healthy control cohort. They observed that anodal tDCS resulted in a larger increase in CBF compared to cathodal tDCS, consistent with existing evidence that the anode tends to enhance the excitability of neurons beneath it [32]. Additionally, Stagg et al. [3] conducted a study in healthy controls, applying tDCS to the left dorsolateral prefrontal cortex (DLPFC). The authors demonstrated that both anodal and cathodal tDCS led to CBF changes in regions anatomically connected to the DLPFC, such as the thalamus and motor cortex. In a previous study [25], we investigated CBF changes induced by tDCS applied to the right DLPFC in a healthy control cohort. We found that anodal tDCS produced a larger increase in CBF compared to cathodal and sham tDCS, affecting areas including the insula, thalamus, occipital cortex, middle cingulate gyrus, and lateral prefrontal cortex.

Acupuncture is an ancient therapy originating from China and has been widely used to treat various conditions. In recent years, it has been increasingly accepted as an effective treatment option for chronic pain [33]. With accumulating evidence, many clinical trials have been published to support using it to manage cLBP [34,35,36,37], which is recommended to be selected initially in a clinical practice guideline from the American College of Physicians [38]. Medicare has also started to cover acupuncture treatment for cLBP (https://www.medicare.gov/coverage/acupuncture; accessed on 27 February 2025) [39]. Nevertheless, some people respond only moderately or not at all to acupuncture [40]. Thus, there is an urgent need to develop new methods to enhance the effectiveness of acupuncture treatment.

Combining tDCS with acupuncture may offer synergistic effects, amplifying the therapeutic benefits of both tDCS and acupuncture for chronic pain [41]. Therefore, in this study, we investigated the neural mechanisms and the modulatory effects of tDCS with or without acupuncture on chronic low back pain (cLBP) using pulsed continuous arterial spin labeling (pCASL) imaging. The tDCS was applied bilaterally to the motor cortex (M1), and the treatment consisted of six sessions over the course of one month. Participants were randomized into four groups based on tDCS and acupuncture treatments. We assessed changes in CBF between pre- and post-treatment for each group. To increase statistical power, we also combined the tDCS or acupuncture groups (real vs. sham) to focus on the overall effects of tDCS or acupuncture treatments. We hypothesized that (1) multiple sessions of real tDCS applied to M1 or real acupuncture would induce distinct changes in CBF compared to sham interventions, and (2) these modulations in CBF may involve key brain regions associated with pain processing and modulation, including the anterior cingulate gyrus, insula, and motor cortex.

## 2. Materials and Methods

### 2.1. Participants

A total of 116 individuals with cLBP were recruited at Massachusetts General Hospital (MGH) between November 2020 and November 2023. Of these, 21 participants were excluded due to scheduling conflicts, and 8 were removed from the analysis due to poor quality or incomplete MRI data. The final cohort included 87 participants (age: 41.06 ± 12.4 (mean ± SD); gender = 43 females, 44 males).

This study received approval from the MGH Institutional Review Board, and all participants provided informed consent prior to enrollment. Participants were randomly assigned to one of four groups: (1) real tDCS with real acupuncture; (2) real tDCS with sham acupuncture; (3) sham tDCS with real acupuncture; (4) sham tDCS with sham acupuncture.

Inclusion criteria were as follows: (1) adults aged 18–65 years; (2) diagnosed with cLBP (defined as low back pain lasting longer than 6 months) by the referring physician; (3) a minimum pain score of 4/10 on the 11-point Numeric Rating Scale (NRS) for low back pain; (4) ability to read and understand English. Exclusion criteria included the following: (1) history of epilepsy; (2) specific underlying causes of back pain (e.g., cancer, fractures, spinal stenosis, infections); (3) complex back conditions (e.g., previous back surgery, ongoing medicolegal issues); (4) intention to undergo surgery during the study period; (5) history of cardiovascular, respiratory, or neurological conditions that, in the investigator’s judgment, would pose significant risks to participation; (6) contraindications to MRI (e.g., pacemakers, metal implants, pregnancy, claustrophobia, inability to remain still during fMRI scans); (7) history of significant medical or psychiatric disorders; (8) substance abuse or dependence history.

### 2.2. Experimental Procedure

Each participant completed a total of 8 sessions. The first session was a behavioral assessment, where participants filled out pain questionnaires and were trained in Quantitative Sensory Testing (QST) using heat and mechanical stimuli.

Sessions 2 through 7 were treatment sessions, where participants about 20 mins of tDCS (real or sham) combined with acupuncture (real or sham, about 30 mins), as determined by their assigned group. The treatment protocol consisted of six sessions over a one-month period, with treatments administered twice a week during the first two weeks and once a week during the last two weeks. 

For real acupuncture, the acupoints utilized include Yaoyangguan (GV03), Shenshu (BL23), Weizhong (BL40), Taixi (KI03), and Ashi acupoints. For the sham acupuncture, validated Streitberger non-penetrating sham acupuncture needles were applied to non-acupoints located on the lower back and leg.

The final session (number 8) involved a post-treatment assessment, which included clinical evaluations and behavioral measures. Additionally, QST measurements were repeated using heat and mechanical pain stimuli. Participants were debriefed at the conclusion of the session.

MRI sequences were acquired during sessions 2 (before starting the treatment) and 8 (after the completion of treatment). The NRS was collected to measure the average low back pain intensity over the past seven days at baseline and at the end of the treatment.

**Figure 1:**
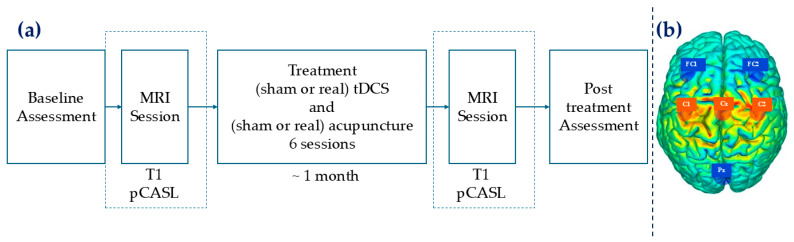
(**a**) Workflow of the experimental procedure. Each participant completed 8 sessions in total. The first and last sessions involved behavioral assessments, where pain intensity was measured using the NRS. Participants underwent two MRI sessions: one before the start of tDCS treatment and another after completing the final treatment session. The treatment phase lasted for 6 sessions over approximately 1 month. (**b**) High-definition anodal tDCS configuration. Anode electrodes are in orange and return electrodes are in blue. Orange dish line represents the stimulation area.

The workflow of the experimental design is reported in Figure 1a.

### 2.3. tDCS Administration

tDCS was administered to the bilateral M1, with participants randomly assigned to either the anodal or sham tDCS group. The tDCS stimulation modes were pre-programmed according to the participants’ randomization in the device system, ensuring blinding for both operators/analysts and participants. For each tDCS session, stimulation was delivered at 2 mA for 20 min using the StarStim system (Neuroelectrics, Barcelona, Spain). Electrodes were positioned over the following locations: Fc_1_, Fc_2_, C_1_, C_2_, C_z_, and P_z_ for both stimulation conditions. In the sham tDCS condition, the current was applied only for 15 s at the beginning and end of the 20 min session to induce a brief tingling sensation, simulating the onset and offset of real stimulation. The electrode impedances were maintained below 10 kΩ throughout all sessions. High-definition tDCS (HD-tDCS) was used to stimulate the bilateral primary motor cortices simultaneously (see Figure 1b) with the following current for each electrode: C_1_ 1500 mA, C_2_ 1500 mA, and C_z_ 999 mA, F_C1_ −1134 mA, F_C2_ −1500 mA, and P_z_ −1365 mA.

Acupuncture treatments were administered by licensed acupuncturists with a minimum of five years of experience. For the active acupuncture treatment, seven acupuncture points commonly used for cLBP were targeted: Du 3, Bladder 23 (bilateral), Bladder 40 (bilateral), and Kidney 3 (bilateral), located on the lower back and legs [42,43]. Additionally, 1–3 ashi (tender) points bilaterally on the lower back and legs were also included. Each session lasted approximately 30 min, with needle stimulation through twirling at the 10 min mark and again just before needle removal. For the sham acupuncture group, Streitberger non-penetrating needles [44] were placed at non-acupuncture sites [45,46]. Needles were inserted and rotated until the participant reported a sensation. We chose to place Streitberger needles at non-acupoints based on a study by Cherkin et al. [42] which demonstrated that using “sham treatment” on real acupuncture points might produce a therapeutic effect due to the stimulation of real acupoints. Thus, we chose to use non-acupoints for the sham treatment [47].

### 2.4. MRI Acquisition

MRI scans were conducted at the MGH Martinos Center for Biomedical Imaging using a 32-channel radiofrequency head coil on a 3T Siemens scanner. Structural brain imaging was performed with a T1-weighted three-dimensional multi-echo magnetization-prepared rapid gradient-echo (MPRAGE) sequence (voxel size: 1 × 1 × 1 mm^3^, repetition time: 2500 ms, echo time: 1.69 ms, slice thickness: 1 mm, flip angle: 7°, 176 slices). To obtain perfusion-weighted images, pCASL was employed using a 2D gradient echo planar imaging sequence (echo time: 15 ms, repetition time: 3800 ms, flip angle: 90°, slice thickness: 5 mm), with 92 volumes acquired in total.

Both pCASL and T1 scans were performed at baseline (prior to treatment initiation) and following the completion of treatment (session 8).

### 2.5. MRI Preprocessing

pCASL data were preprocessed using tools from the FMRIB’s Software Library (FSL, www.fmrib.ox.ac.uk/fsl; accessed on 30 September 2024) version 6.0.1. The standard preprocessing pipeline included the following steps: (i) motion correction via MCFLIRT; (ii) removal of non-brain tissue using the Brain Extraction Tool (BET); (iii) spatial smoothing with a Gaussian kernel (5 mm full width at half-maximum); (iv) normalization to the Montreal Neurological Institute (MNI) standard brain template. Perfusion-weighted time series were generated by the pairwise subtraction of control and label images.

### 2.6. Statistical Analysis

To assess the changes in perfusion or CBF induced by tDCS and acupuncture, a mixed-effects model was applied to analyze group-level differences in perfusion across the entire brain: (i) pre- vs. post-treatment (session 2 vs. session 8) within each of the four groups; (ii) pre-tDCS vs. post-tDCS (session 2 vs. session 8) for sham and real tDCS groups respectively; (iii) comparisons of pre- vs. post-tDCS changes between the sham and real tDCS groups; (iv) pre vs. post acupuncture for sham and real acupuncture groups respectively; (v) comparisons of pre vs. post acupuncture changes between sham and real acupuncture groups. We used mixed-effects higher-level modeling using FLAME 1 (FMRIB’s Local Analysis of Mixed Effects) from the FEAT (FMRI Expert Analysis Tool) version 6.0.1, part of FSL, to account for session/subject variability in estimations. Depending on the comparison, a general linear model (GLM) was built that included explanatory variables (EVs) for each of the experimental groups being compared as well as EVs to account for paired comparisons between pre- and post-intervention for each subject using a full model set up in FEAT. Resulting Z-Statistic images were thresholded using a non-parametric, cluster-based approach to identify regions exhibiting significant changes in perfusion. Clusters were defined with a Z-score threshold of Z > 1.64 and a corrected cluster *p*-value of 0.05.

## 3. Results

### 3.1. Demographic Characteristics

ASL data from 74 participants were included for data analysis: 20 received real acupuncture with real tDCS, 19 received sham acupuncture with real tDCS, 18 received real acupuncture with sham tDCS, and 17 received sham acupuncture with sham tDCS.

No differences in age and gender were found between the four groups (Table 1).

The real tDCS group consisted of 39 subjects, and the sham tDCS group consisted of 35 subjects. 38 subjects received real acupuncture treatment, and 36 subjects received sham acupuncture.

No differences in age, gender, and acupuncture treatment were found between real and sham groups in both tDCS and acupuncture. The demographic characteristics can be found in Table 1.

### 3.2. Within-Group Arterial Spin Labeling Analysis Results

#### 3.2.1. Sham Acupuncture and Sham tDCS

In the contrast between pre- vs. post-tDCS treatment, sham tDCS and sham acupuncture led to a decrease in the CBF in the left lateral occipital cortex and left middle/superior frontal gyrus. No significant results were found in the opposite contrast.

#### 3.2.2. Sham Acupuncture and Real tDCS

In the real tDCS and sham acupuncture group, we found a decreased CBF between pre- and post-treatment in the bilateral occipital cortex and precuneus. No significant results were found in the opposite contrast.

#### 3.2.3. Real Acupuncture and Sham tDCS

No significant results between pre- vs. post-treatment were found for subjects who received sham tDCS and real acupuncture at the threshold we set.

#### 3.2.4. Real Acupuncture and Real tDCS

In the contrast between pre- vs. post-tDCS treatment, real tDCS with and real acupuncture group showed an increase in the CBF post-treatment in the right superior temporal gyrus, right supramarginal gyrus, right angular gyrus, and right planum temporale. No other significant results were found in the opposite contrast.

**Figure 2:**
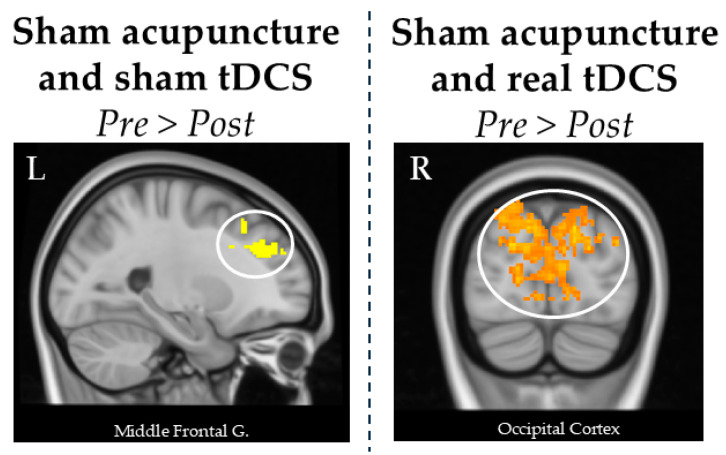
Significant CBF clusters found in the statistical analysis of the four different groups. Sham acupuncture and sham tDCS led to a decrease in the left middle frontal gyrus. The real tDCS and sham acupuncture group was associated with decreased CBF in the bilateral occipital cortex. All these brain regions are shown in the circles of the figures.

These results are reported in Table 2 and Figure 2.

### 3.3. tDCS Arterial Spin Labeling Analysis Results

#### 3.3.1. Real tDCS

By comparing the perfusion data between baseline and post-treatment, we found that real tDCS led to reduced CBF post-treatment in the bilateral supracalcarine and intracalcarine cortex, bilateral lateral occipital cortex, bilateral cuneus, bilateral precuneus, left hippocampus, and left parahippocampal gyrus/posterior cingulate cortex.

**Figure 3:**
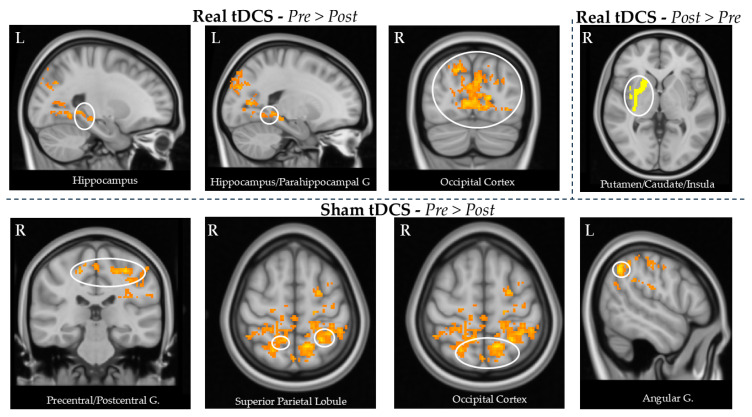
CBF changes in real and sham tDCS between pre- and post-treatment. Real tDCS led to decreased CBF after the treatment in the left hippocampus and parahippocampal gyrus, and in the bilateral occipital cortex. In addition, real tDCS was associated with increased CBF after the treatment in the right putamen/caudate/insula. Sham tDCS was associated with decreased CBF in the right precentral/postcentral gyrus, left superior parietal gyrus, left occipital gyrus, and left angular gyrus. All these brain regions are shown in the circles of the figures.

In the opposite contrast, there was a significant increase in CBF observed in the right putamen, caudate, and insula. These results are reported in Table 3 and Figure 3.

#### 3.3.2. Sham tDCS

In the contrast between pre- and post-tDCS treatment, the sham group showed a decrease in the CBF post-treatment in the bilateral superior parietal lobule, bilateral precuneus, left angular gyrus, bilateral precentral and postcentral gyri, and bilateral occipital cortex.

No other significant results were found in the opposite contrast. These results are reported in Table 3 and Figure 3.

#### 3.3.3. Between-Groups Comparison

The between-group comparisons of the differences in pre- and post-tDCS showed that real tDCS was associated with greater increase perfusion in the bilateral anterior cingulate cortex, left superior frontal gyrus, right occipital cortex, right superior frontal/postcentral gyrus, right putamen/caudate, and right insula. No significant results were found in the opposite contrast. 

**Figure 4:**
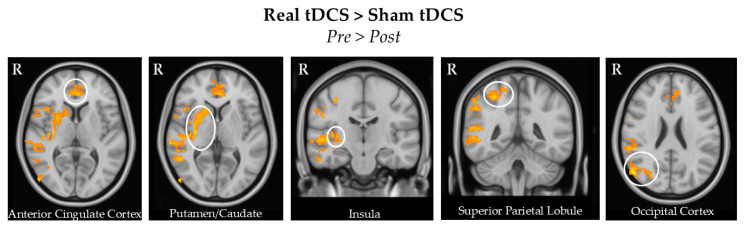
Comparing pre- and post-treatment differences between the real and sham tDCS groups. The difference highlights regions where the post-treatment minus pre-treatment change in CBF is greater in the real tDCS group compared to the sham group. We found that the real tDCS led to a larger increase in the bilateral anterior cingulate cortex, right putamen/caudate, right insula, right superior parietal lobule, and right occipital cortex. All these brain regions are shown in the circles of the figures.

These results can be found in Table 4 and Figure 4.

### 3.4. Acupuncture Arterial Spin Labeling Analysis Results

#### 3.4.1. Real Acupuncture

Comparison of the perfusion data between baseline and post-treatment in the real acupuncture group revealed a reduction in CBF in the bilateral intracalcarine cortices, bilateral cuneus, bilateral precuneus, bilateral hippocampus, left posterior cingulate gyrus, and left middle temporal gyrus.

**Figure 5:**
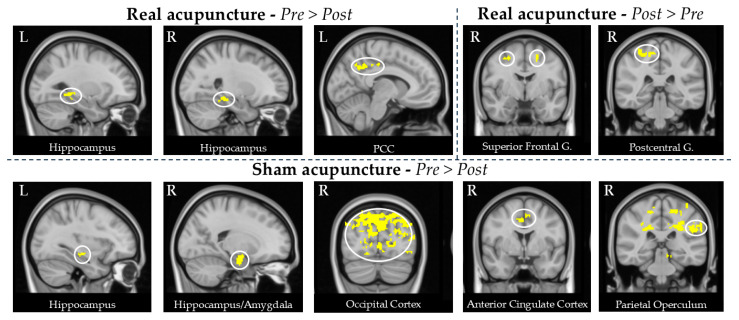
Statistical analysis of the CBF changes for the real and sham acupuncture groups. Real acupuncture led to decreased CBF in the bilateral hippocampus and left posterior cingulate cortex (PCC). In addition, increased CBF in the bilateral superior frontal gyrus and right postcentral gyrus were detected. Sham acupuncture was associated with decreased CBF in the bilateral hippocampus, bilateral occipital cortex, bilateral anterior cingulate cortex, and left parietal operculum. All these brain regions are shown in the circles of the figures.

In the opposite contrast, we observed an increase in CBF in the right postcentral gyrus, right superior parietal lobule, right middle frontal gyrus, and bilateral superior frontal gyrus. These results are reported in Table 5 and Figure 5.

#### 3.4.2. Sham Acupuncture

In the comparison between pre- and post-treatment, sham acupuncture led to a decreased CBF in the bilateral occipital cortex, bilateral cuneal cortex, bilateral precuneus, bilateral hippocampus, right amygdala, bilateral anterior cingulate gyrus, bilateral frontal pole, bilateral middle frontal gyrus, bilateral precentral gyrus, bilateral planum polare, right supplementary motor cortex, and left parietal operculum. No significant results were found in the other contrast. 

Results are reported in Table 5 and Figure 5.

#### 3.4.3. Between Groups Comparison

The between-group comparisons of the differences in pre- and post-acupuncture showed that real acupuncture treatment led to a greater increase in perfusion in the bilateral precentral and postcentral gyri, left superior frontal gyrus, bilateral middle frontal gyrus, and left lateral occipital cortex.. No significant results were found in the opposite contrast. 

**Figure 6:**
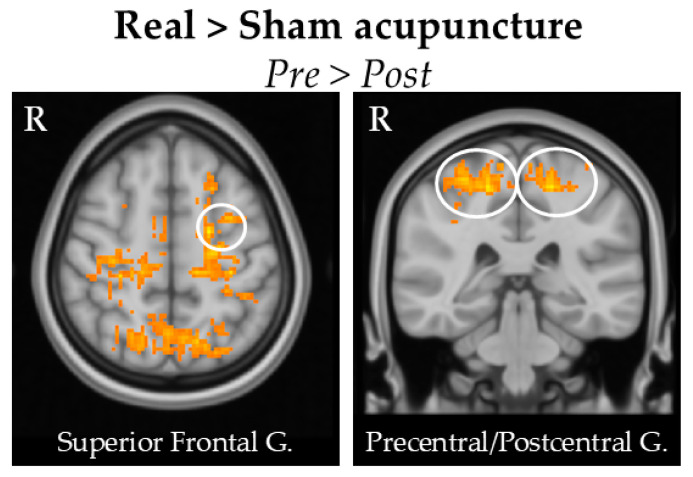
Comparing pre- and post-treatment differences between the real and sham acupuncture groups. The difference highlights regions where the post-treatment minus pre-treatment change in CBF is greater in the real acupuncture group compared to the sham group. We found that real acupuncture led to a larger increase in the left superior frontal gyrus and bilateral precentral gyrus. All these brain regions are shown in the circles of the figures.

These results can be found in Table 6 and Figure 6.

### 3.5. Clinical Outcome Summary

Both real and sham tDCS showed a significant decrease in pain after the treatments (real, *p* = 0.002; sham, *p* = 0.004). However, no significant difference was found in the pain intensity decrease between the groups (*p* = 0.76; [mean ± standard deviation] real = −0.82 ± 1.57; sham = −0.94 ± 1.79).

Also, both real and sham acupuncture groups reported a significant pain decrease (real, *p* = 0.0001; sham, *p* = 0.03). No significant difference between real and sham acupuncture was found in the pain intensity decrease (*p* = 0.19; [mean ± standard deviation] real = −1.13 ± 1.65, sham = −0.61 ± 1.68).

Within group comparisons showed that the sham acupuncture and sham tDCS group as well as the sham acupuncture and real tDCS group did not show a significant pain decrease after the treatment (*p* = 0.21 and 0.09 respectively). Real acupuncture and sham tDCS reported a significant pain decrease (*p* = 0.007) as well as real acupuncture and real tDCS (*p* = 0.009).

## 4. Discussion

In this study, we investigated how multiple sessions of tDCS and acupuncture influence CBF in individuals with cLBP. To increase statistical power, we combined the four groups into two main categories based on tDCS (real and sham) and acupuncture (real and sham) randomization, allowing for a more focused comparison between the active and sham conditions for both tDCS and acupuncture while accounting for each group independently in our statistical model to control for any differences between the groups. We found that comparisons of pre- and post-treatment CBF between the real and sham tDCS groups revealed that post treatment changes in response to real tDCS were larger than sham tDCS in the bilateral anterior cingulate cortex, left superior frontal gyrus, right occipital cortex, right superior frontal/postcentral gyrus, right putamen and caudate, and right insula. A comparison between real and sham acupuncture showed that real treatment led to increased CBF in the bilateral precentral and postcentral gyri, left superior frontal gyrus, bilateral middle frontal gyrus, left lateral occipital cortex, bilateral precuneus, and right supplementary motor cortex. Our findings suggest that the effects of tDCS and acupuncture on brain activity are mode-dependent and may reflect alterations in the neural circuits underlying chronic pain.

### 4.1. Brain Regions Associated with tDCS

The findings of our study suggest that both real and sham tDCS at the bilateral M1 led to a decrease in CBF in the bilateral precuneus and occipital cortex. The precuneus, a key region of the default mode network [48], plays a central role in cognitive functions such as consciousness, memory, self-reflection, and integration of sensory stimuli [49,50].

Previous research has highlighted the active involvement of the precuneus in chronic pain [50,51,52,53,54] and its associated changes in CBF during prolonged pain states [48,55,56]. Taken together, these studies suggest that the precuneus is an important neural hub in pain processing, particularly in the modulation of cognitive functions within the default mode network [50]. Iwabuchi et al. [48] observed significant patterns of relative hypoperfusion in the default mode network, with the precuneus as a central hub, in individuals with chronic knee pain. This may be an adaptive coping mechanism to distract from the ongoing pain in these patients.

We also observed a decrease in CBF of occipital regions in both real and sham tDCS groups. This result aligns with our previous finding [25] where we showed that real tDCS applied to the right DLPFC can modulate CBF in the occipital cortex in healthy controls. Traditionally, the occipital cortex has been linked to visual processing, but recent evidence suggests it may also play a role in cLBP, identifying visual network disruptions in cLBP patients [57,58]. A previous study has shown that noxious stimuli, such as heat pain, lead to decreased fMRI signals in the occipital cortices [59]. Moreover, machine-learning-based analysis found that alterations in gray matter density in the visual cortex and temporal lobe can differentiate cLBP patients from healthy controls [57]. Together, these findings suggest that the occipital cortex may be involved in the modulation of pain, although its precise role remains unclear.

Additionally, the occipital cortex may interact with pain-related regions, such as the accumbens and thalamus, which could influence how pain and sensory stimuli are perceived and processed [60,61]. These interactions highlight the complex relationship between visual processing regions and pain modulation, warranting further investigation into their potential role in chronic pain conditions. Building on these insights, our results suggest that the decreased CBF observed in the bilateral precuneus and occipital cortex following the repeated real and sham tDCS at M1 may reflect an adaptive mechanism, enabling individuals with cLBP to more effectively cope with ongoing nociceptive input. 

Moreover, we found that real tDCS could modulate the CBF of the left hippocampus and parahippocampal gyrus. The hippocampus and parahippocampal gyrus play a pivotal role in chronic pain [62]. Both of these regions are part of the limbic system and are strongly involved in the cognitive and memory-related aspects of pain, as well as in the emotional and affective aspects of pain perception [27,63]. In fact, some studies reported their activation in experimental settings involving painful stimuli [27,63,64,65]. Alterations of CBF in the hippocampus and parahippocampal gyrus have been detected in a chronic pain study [62] in which individuals with ongoing pain in osteoarthritis showed increased CBF in these areas in comparison to healthy controls, suggesting the involvement of these regions in pain maintenance. As such, our results showing a reduction in CBF in these areas post-real tDCS hold potential therapeutic promise. By modulating CBF in these regions, real tDCS may influence the way chronic pain is perceived, remembered, and emotionally processed (factors that are often implicated in chronic pain maintenance [62]). This suggests that tDCS could have therapeutic potential not only in modulating sensory pain processing, but also in altering pain-related memories and emotional responses, which are central to the experience of chronic pain.

In addition, our results showed that real tDCS applied at M1 led to increased CBF in the right basal ganglia (putamen and caudate) and insula cortex. The basal ganglia [66] play a crucial role in pain processing [53,67,68,69], while the insula serves as an important cortical relay center for pain and interoception [70]. Previous studies have suggested that modulation or activation of both the basal ganglia and the insula may contribute to pain relief [71,72,73]. In this context, the CBF modulation of these crucial pain regions may play an important role in pain modulation and relief. Specifically, increased CBF in the right basal ganglia and insula following tDCS application may enhance neural activity and connectivity within these areas, potentially leading to improved pain perception regulation. Therefore, modulation of CBF in these areas via real tDCS could represent a promising therapeutic approach for pain management, highlighting the potential for non-invasive neuromodulation to influence complex pain networks.

Considering the sham tDCS, repeated treatment sessions led to decreased CBF between pre- and post-tDCS in the bilateral superior parietal lobule, left angular gyrus, and bilateral precentral and postcentral gyri.

The superior parietal lobule is a critical component of the dorsal attention network, playing a pivotal role in integrating various sensory inputs [74,75]. This region has been shown to be involved in the processing and coordination of spatial attention, which is essential for guiding adaptive behaviors in response to environmental stimuli [74]. Some studies have demonstrated the superior parietal lobule’s involvement in chronic pain, highlighting its altered activity in chronic pain states [74,75,76,77]. In addition, alterations in the superior parietal lobule activity have been shown in cLBP [75,77]. These findings underscore the involvement of the superior parietal lobule in both sensory processing and the neural mechanisms underlying chronic pain, suggesting that the brain’s attention systems may be altered in the context of persistent pain [75]. Thus, the decreased CBF observed in the superior parietal lobule post-treatment in our study may reflect the ability of sham tDCS to promote an adaptive response to salient stimuli most likely through placebo analgesic mechanisms.

The precentral and postcentral gyri are essential brain regions within the sensorimotor network, which plays a pivotal role in processing sensory information and facilitating motor planning [78,79]. Furthermore, research has demonstrated that the precentral and postcentral gyri are also engaged in the maintenance of pain, underscoring their importance not only in sensory and motor processes but also in the complex mechanisms of chronic pain [78,80,81]. Growing evidence suggests that the sensorimotor network may be implicated also in the processes underlying placebo analgesia, with some studies indicating that this network could play a crucial role in modulating pain perception [82,83,84]. The decreased CBF observed in the precentral and postcentral gyri suggests that sham tDCS may modulate the sensorimotor network, which is associated with placebo analgesia, influencing pain perception and experience.

To summarize, our findings identified shared brain regions, such as the precuneus and occipital cortex, involved in the mechanisms underlying both real and sham tDCS, along with distinct brain networks specific to each tDCS condition. These results offer strong evidence that real and sham tDCS may activate different neurobiological processes, while also suggesting potential overlaps, particularly in relation to cognitive and attention-related regulation of pain.

### 4.2. Brain Regions Associated with Acupuncture

We observed that both real and sham acupuncture can reduce CBF in the hippocampus, precuneus, and occipital cortex. Research indicates that changes in hippocampal CBF may affect how chronic pain is perceived, emotionally processed, and involved in acupuncture treatment of chronic pain [85]. Similar to tDCS, the decreased CBF in the bilateral precuneus and occipital cortex might represent an adaptive mechanism, helping individuals with cLBP to better manage ongoing nociceptive input [58]. These findings suggest that both real and sham acupuncture may influence the modulation of pain-related memories, emotional responses, and cognitive processes, which represent key factors in the experience of cLBP.

We also found that real acupuncture treatment is associated with CBF changes in several important brain areas such as the posterior cingulate gyrus, superior parietal lobule, postcentral gyrus, and frontal areas. The posterior cingulate gyrus, a key region of the default mode network, is associated with cognitive and attention tasks [86]. In addition, it plays a pivotal role in cLBP in processing negative emotions [87]. The observed reductions in CBF in the hippocampus and posterior cingulate gyrus suggest that real acupuncture may modulate the cognitive and emotional aspects of pain. Such modulation may have implications for both the sensory and emotional dimensions of pain, suggesting that acupuncture may provide important therapeutic benefits in managing both the physical and psychological aspects of chronic pain.

Moreover, the observed changes in CBF in the postcentral gyrus further support the potential of real acupuncture in modulating pain perception. The postcentral gyrus is a key area involved in the somatosensory processing of pain stimuli [80,81,82], and changes in CBF in this region may indicate acupuncture’s ability to influence how pain is processed at the cortical level. Together, these findings highlight the complex and multifaceted role of acupuncture in modulating both the sensory and emotional components of chronic pain.

Real acupuncture also led to an increase in CBF in the middle and superior frontal gyrus, brain regions involved in higher cognitive functions such as attention, decision making, and emotional regulation [88,89]. These areas are also integral to pain processing, particularly in the modulation of pain perception and the integration of cognitive and emotional responses to pain [90,91]. We can speculate that the increased CBF in these frontal regions suggests that real acupuncture may enhance cognitive control over pain, increasing attentional focus away from pain or enhancing the brain’s capacity to manage pain-related distress.

We found that sham acupuncture was associated with CBF decreases in the anterior cingulate gyrus, precentral gyrus, and parietal operculum. The anterior cingulate gyrus is a key region involved in placebo analgesia, as highlighted in several studies [82,92]. It is also implicated in both pain processing and emotional regulation [93,94]. We speculate that the observed decrease in CBF in the anterior cingulate gyrus following sham acupuncture may reflect the activation of cognitive and emotional pathways typically involved in the placebo response.

The precentral gyrus and parietal operculum are involved in the sensory processing of pain [4,82,95]. Additionally, the parietal operculum contains the secondary somatosensory cortex (S2), which is essential for identifying and processing various sensory stimuli from the body, including pain [96,97]. In a previous study [4], we found that the parietal operculum may play a significant role in placebo analgesia, likely through its connections with the dorsal attention network.

In summary, our findings revealed that both real and sham acupuncture activate common brain regions, such as the hippocampus and occipital cortex, alongside distinct brain networks specific to each intervention. These results provide compelling biological evidence that real and sham acupuncture engage different neurobiological mechanisms, while also suggesting potential overlaps, particularly in the context of emotional and cognitive regulation of pain.

### 4.3. Limitation

The findings of this study should be interpreted with some limitations. First, the relatively small sample size may limit generalizability. Second, the experimental design used for tDCS application differs from other studies [41,98]. Specifically, in our study, tDCS was applied six times over the course of one month, which may contrast with other protocols where tDCS is typically applied more frequently or for a longer duration [41,98]. Moreover, the absence of a long-term follow-up in this study limits our understanding of the lasting effects of tDCS. Future research should address the durability of this intervention over time to better assess its long-term impact. Additionally, this study focused on a specific population, cLBP, and this may limit the applicability of our results to other pain conditions.

## 5. Conclusions

In this study, we examined the effects of tDCS and acupuncture on CBF in individuals with cLBP. Our findings reveal distinct patterns of CBF changes associated with each treatment modality, with some shared regions (the hippocampus and occipital cortex for both real and sham acupuncture, and the precuneus and occipital cortex for both real and sham tDCS). These results suggest that the effects of repeated tDCS and acupuncture on brain activity are mode-dependent, yet may be interconnected, particularly in relation to the emotional processing of pain during real and sham acupuncture, and cognitive regulation during both real and sham tDCS and acupuncture. Together, our study underscores the complexity of pain modulation and highlights the potential of both tDCS and acupuncture as non-pharmacological interventions for chronic pain management.

## Figures and Tables

**Table 1:** Demographic characteristics for both sham and real treatments in tDCS and acupuncture groups.

Groups					
	Sham Acu and Sham tDCS	Sham Acu and Real tDCS	Real Acu and Sham tDCS	Real Acu and Real tDCS	P
Number of Subjects	17	19	18	20	
Age (mean ± std)	41.0 ± 12.7	38.1 ± 12.1	40.3 ± 12.7	44.0 ± 13.9	0.55 ^#^
Gender (F/M)	7/10	10/9	10/8	12/8	0.70 ^$^
tDCS					
	Sham		Real		P
Number of Subjects	35		39		
Age (mean ± std)	40.7 ± 12.5		41.1 ± 13.2		0.88 *
Gender (F/M)	17/18		22/17		0.66 ^$^
Acupuncture (Y/N)	18/17		20/19		0.98 ^$^
Acupuncture					
	Sham		Real		P
Number of Subjects	36		38		
Age (mean ± std)	39.5 ± 12.3		42.3 ± 13.3		0.35 *
Gender (F/M)	17/22		19/16		0.5 ^$^
tDCS (Y/N)	19/17		20/18		1 ^$^

* Two-sample *t* test; ^$^ Chi-squared test; ^#^ ANOVA; F = female; M = male; Y = yes; N = no.

**Table 2:** Within group pCASL comparison results across the four groups: sham acupuncture and sham tDCS, sham acupuncture and real tDCS, real acupuncture and sham tDCS, and real acupuncture and real tDCS.

Comparison	Region (Peak Cluster)	Peak MNI Coordinates (x, y, z)		
Sham acupuncture and sham tDCS				
Pre- vs. post-tDCS	Left Lateral Occipital Cortex	−52	−60	44
	Left Middle Frontal Gyrus	−24	36	30
Post- vs. pre-tDCS	No voxel survived to the threshold			
Sham acupuncture and real tDCS				
Pre- vs. post-tDCS	Right Occipital Cortex	18	−78	48
	Right Precuneus	8	−68	30
Post- vs. pre-tDCS	No voxel survived to the threshold			
Real acupuncture and sham tDCS				
Pre- vs. post-tDCS	No voxel survived to the threshold			
Post- vs. pre-tDCS	No voxel survived to the threshold			
Real acupuncture and real tDCS				
Pre- vs. post-tDCS	No voxel survived to the threshold			
Post- vs. pre-tDCS	Right Superior Temporal Gyrus	54	−18	−4
	Right Supramarginal Gyrus	50	−40	4
	Right Angular Gyrus	50	−52	4
	Right Planum Temporale	56	−18	2

**Table 3:** pCASL results between pre- and post-treatment in real and sham tDCS groups.

Comparison	Region (Peak Cluster)	Peak MNI Coordinates (x, y, z)		
Real tDCS groups				
Pre > post	Right Supracalcarine Cortex	4	−82	8
	Right Lateral Occipital Cortex	16	−78	42
	Right Intracalcarine Cortex	6	−68	8
	Right Cuneus/Precuneus	10	−70	24
	Left Intracalcarine Cortex	−16	−74	10
	Left Lateral Occipital Cortex	−46	−54	−8
	Left Hippocampus	−30	−32	−4
	Left Hippocampus/Parahippocampal Gyrus	−20	−40	−4
Post > pre	Right Putamen/Caudate/Insula	30	0	0
Sham tDCS groups				
Pre > post	Left Occipital Cortex	−18	−58	66
	Left Precuneus	−12	−56	58
	Left Angular Gyrus	−52	−58	42
	Right Precentral/Postcentral gyrus	18	−30	54
	Left Superior Parietal Lobule	−26	−50	58
Post > Pre	No voxels survived to the threshold			

**Table 4:** pCASL results in the differences between real and sham tDCS.

Comparison	Region (Peak Cluster)	Peak MNI Coordinates (x, y, z)		
Real tDCS > Sham tDCS				
Pre > post	Left Anterior cingulate cortex	0	40	6
	Left Superior frontal gyrus	−6	26	52
	Right Occipital Cortex	54	−70	22
	Right Superior Parietal Lobule	32	−44	62
	Right Putamen/Caudate	30	0	0
	Right Insula	32	−16	4
Sham tDCS > Real tDCS				
Pre > post	No voxels survived to the threshold			

**Table 5:** pCASL results between pre- and post-treatment in real and sham acupuncture groups.

Comparison	Region (Peak Cluster)	Peak MNI Coordinates (x, y, z)		
Real acupuncture groups				
Pre > post	Left Intracalcarine Cortex	−12	−66	8
	Left Middle Temporal Gyrus	−52	−16	−20
	Left Hippocampus	−28	−38	−6
	Left Posterior Cingulate Cortex (PCC)	−8	−44	36
	Right Hippocampus	26	−32	−14
Post > Pre	Right Postcentral Gyrus	30	−34	56
	Right Superior Parietal Lobule	26	−50	66
	Right Middle Frontal Gyrus	36	36	18
	Left Superior Frontal Gyrus	−20	−4	56
	Right Superior Frontal Gyrus	26	−4	50
Sham acupuncture groups				
Pre > Post	Left Occipital Cortex	−28	−72	52
	Left Cuneus Cortex	−4	−96	14
	Left Hippocampus	−38	−24	−10
	Right Anterior Cingulate Cortex	2	2	44
	Right Frontal Pole	46	40	0
	Right Hippocampus/Amygdala	20	−10	−24
	Right Middle Frontal Gyrus	34	36	34
	Right Precentral Gyrus	44	0	38
	Right Planum Polare	44	−10	−10
	Right Supplementary Motor Cortex	6	−18	54
	Left Parietal Operculum	−54	−35	27
	Left Frontal Pole	−44	40	4
	Left Precuneus	−10	−54	16
Post > Pre	No voxels survived to the threshold			

**Table 6:** pCASL results in the differences between real and sham acupuncture.

Comparison	Region (Peak Cluster)	Peak MNI Coordinates (x, y, z)		
Real acupuncture > Sham acupuncture				
Pre > post	Left Superior Frontal/Precentral Gyrus	−20	−6	54
	Right Precentral/Postcentral Gyrus	18	−34	56
	Left Postcentral Gyrus	−18	−34	56
	Left Middle Frontal Gyrus	−32	0	48
	Left Lateral Occipital Cortex	−14	−66	48
Sham acupuncture > Real acupuncture				
Pre > post	No voxels survived to the threshold			

## Data Availability

The data that support the findings of this study are available from the corresponding author upon reasonable request due to privacy and ethical reasons.

## Abbreviations

tDCS	Transcranial direct current stimulation
cLBP	Chronic low back pain
pCASL	Pulsed continuous arterial spin labeling
ASL	Arterial spin labeling
MRI	Magnetic resonance imaging
CBF	Cerebral blood flow
M1	Motor cortex
QST	Quantitative Sensory Testing
MGH	Massachusetts General Hospital
FLAME 1	FMRIB’s Local Analysis of Mixed Effects
FEAT	FMRI Expert Analysis Tool
EV	Explanatory variables
NRS	Numeric Rating Scale
GLM	General linear model
BOLD	Blood oxygenation level-dependent
